# Comprehensive analysis of anoikis-related long non-coding RNA immune infiltration in patients with bladder cancer and immunotherapy

**DOI:** 10.3389/fimmu.2022.1055304

**Published:** 2022-11-25

**Authors:** Yao-Yu Zhang, Xiao-Wei Li, Xiao-Dong Li, Ting-Ting Zhou, Chao Chen, Ji-Wen Liu, Li Wang, Xin Jiang, Liang Wang, Ming Liu, You-Guang Zhao, Sha-dan Li

**Affiliations:** ^1^ Department of Urology, The General Hospital of Western Theater Command, Chengdu, China; ^2^ Department of Urology, The Affiliated Hospital of Southwest Medical University, Luzhou, China; ^3^ Department of Urology, Xuanhan Chinese Medicine Hospital, Dazhou, China

**Keywords:** bladder cancer, lncRNA, anoikis, prognostic model, bioinformatics, immune status

## Abstract

**Background:**

Anoikis is a form of programmed cell death or programmed cell death(PCD) for short. Studies suggest that anoikis involves in the decisive steps of tumor progression and cancer cell metastasis and spread, but what part it plays in bladder cancer remains unclear. We sought to screen for anoikis-correlated long non-coding RNA (lncRNA) so that we can build a risk model to understand its ability to predict bladder cancer prognosis and the immune landscape.

**Methods:**

We screened seven anoikis-related lncRNAs (arlncRNAs) from The Cancer Genome Atlas (TCGA) and designed a risk model. It was validated through ROC curves and clinicopathological correlation analysis, and demonstrated to be an independent factor of prognosis prediction by uni- and multi-COX regression. In the meantime, Kyoto Encyclopedia of Genes and Genomes (KEGG) enrichment analysis, immune infiltration, and half-maximal inhibitory concentration prediction (IC50) were implemented with the model. Moreover, we divided bladder cancer patients into three subtypes by consensus clustering analysis to further study the differences in prognosis, immune infiltration level, immune checkpoints, and drug susceptibility.

**Result:**

We designed a risk model of seven arlncRNAs, and proved its accuracy using ROC curves. COX regression indicated that the model might be an independent prediction factor of bladder cancer prognosis. KEGG enrichment analysis showed it was enriched in tumors and immune-related pathways among the people at high risk. Immune correlation analysis and drug susceptibility results indicated that it had higher immune infiltration and might have a better immunotherapy efficacy for high-risk groups. Of the three subtypes classified by consensus clustering analysis, cluster 3 revealed a positive prognosis, and cluster 2 showed the highest level of immune infiltration and was sensitive to most chemistries. This is helpful for us to discover more precise immunotherapy for bladder cancer patients.

**Conclusion:**

In a nutshell, we found seven arlncRNAs and built a risk model that can identify different bladder cancer subtypes and predict the prognosis of bladder cancer patients. Immune-related and drug sensitivity researches demonstrate it can provide individual therapeutic schedule with greater precision for bladder cancer patients.

## Introduction

As one of the commonest tumors in the urinary tract, bladder cancer grows year by year in terms of both morbidity and mortality (1, 2), and becomes an unrelenting threat to human health. Though only about 25% of patients are diagnosed with bladder wall infiltration or even distant metastases, most of them would die of the disease within two years of diagnosis if left untreated (3, 4). The number of bladder cancer treatment options has increased for the past few years. However, some terminal patients still cannot be effectively treated at the early stage for lack of specific biomarkers (5), and are thus in dire need of effective prognostic prediction biomarkers and new therapeutic targets.

Anoikis, a special type of apoptosis, is initiated when a cell denies interaction with the adjacent extracellular matrix (ECM) (6, 7). ECM includes a network of manifold cytokines that affects cell growth, cell motility and angiogenesis, and they are available to cells through enzymatic digestion and cytoskeletal remodeling (8). It has been reported that anoikis is vital for endometrial carcinoma (9).

Long non-coding RNAs (lncRNAs) are a group of RNAs that are longer than 200 nt but are without protein-coding functions (10). They take an important part in homeostasis and tumorigenesis (11) and serve as tumor markers for early screening, diagnosis, prognosis, and prediction of response to medication (12, 13). lncRNA-related models are of great importance to colon cancer (14), lung adenocarcinoma (15) and pancreatic adenocarcinoma (16), etc. However, studies on the arlncRNAs in bladder cancer prognosis and tumor immune microenvironment (TME) have not been reported so far.

In the present research, we have identified bladder-cancer-related arlncRNAs and developed a risk model that has the potential to guide prognostic prediction and clinical medication.

## Materials and methods

### Information extraction from datasets

Bladder cancer RNA sequencing (RNA-seq) data and clinical data were downloaded from The Cancer Genome Atlas (TCGA) database (https://tcga-data.nci.nih.gov/tcga/), and the inclusion criteria were as follows (1): patients diagnosed with bladder Cancer; and (2) patients with detailed lncRNA and clinical information. After excluding the patients visited less than 30 days, the information of a total of 430 bladder cancer patients was collected.

### Selection of anoikis-related genes and lncRNAs

We obtained 434 Anoikis-related genes (ARGs) (**Supplementary Table S1**) (9) from previously published literature. First, ARGs and lncRNAs filtering were performed *via* Pearson correlation analysis in the condition of |Pearson R| > 0.5 and p<0.001 to get a total of 1109 arlncRNAs. Then, 223 differentially expressed lncRNAs were obtained by filtering the synthetic data matrix through Strawberry Perl V-5.30.0 (https://www.perl.org/) and R software V-4.1.3 according to the criteria of Log2 fold change (FC) >2 and fdrFilter (FDR) < 0.05. Analysis was conducted suing the limma R package (17).

### Creation and validation of the prognosis risk assessment model

First, we screened out the lncRNA associated with the prognosis for patients from lncRNAs by means of uni-COX regression and p<0.05. Next, through last absolute shrinkage and selection operator (LASSO) Cox analysis (18), we discovered seven lncRNAs (**Supplementary Table S2**) and used them to create a risk model based on arlncRNAs. Below is the risk score formula: risk score = ∑[Exp(lncRNA) × coef(lncRNA)]. After that, we randomly divided all samples into a test group and a train group while classifying the bladder cancer patients into a low-risk group and a high-risk group pursuant to the median risk score acquired from the risk model (19). Next, we used the ROC curve,area under the ROC curve (AUC) and the survival curve to validate the accuracy and prognostic value of the model.

### Construction of an anoikis-related model nomogram

According to the clinical data of bladder cancer patients, including, for instance, age, gender, TNM stage, and grade, we used an “rms” R package to draw a nomogram that could assess the one-, three- and five-year overall survival (OS) of patients, and made calibration curves to demonstrate the predictive power of the alignment chart.

### Gene set enrichment analysis

Similarly, bladder cancer patients were classified into a low-risk group and a high-risk group by median risk score. We harnessed gene set enrichment analysis (GSEA) software to look for the differentially expressed Kyoto Encyclopedia of Genes and Genomes (KEGG) pathways in the two groups, and p < 0.05 and FDR < 0.25 were considered statistically significant.

### Analysis of TME and immune checkpoints

To further the analysis of immune cell factors in the high- and low-risk groups, we assessed the relationship between immune cell subpopulations and risk score values through Spearman correlation analysis on TIMER 2.0 (http://timer.cistrome.org/), including CIBERSORT, TIMER, XCELL, QUANTISEQ, MCPcounter, EPIC, and CIBERSORT. The evaluation results are detailed in the bubble chart. With the help of stromal and immune cell scores, we investigated the abundance of immune and stromal cells between cytomes to evaluate the TME differences in the two risk groups. Wilcoxon signed-rank test was implemented to compare the differences, and p < 0.05 was considered remarkable. Subsequently, the bladder-cancer-infiltrating immune cells were scored *via* single-sample GSEA (ssGSEA) and the “GSVA” software package to quantify their relative content. The results are shown in the boxplot. Finally, we compared the immune checkpoint activation between the two risk groups *via* “ggpubr” R package.

### Exploration of model in clinical therapy

The “pRophetic” R package was applied to estimate the half-maximal inhibitory concentration prediction (IC50) of bladder cancer drugs, in the hope of developing drugs that are relevant to the model and may become the candidates for the treatment of bladder cancer.

### Consensus clustering

It was clustered “using ConsensusClusterPlus” (CC) R package in the light of arlncRNA expression. For different subgroups of bladder cancer patients, the “Rtsne” R package was employed to conduct principal component analysis (PCA), T-distributed stochastic neighbor embedding (t-SNE) and Kaplan-Meier survival analysis, and immunocorrelation analysis, and compare prognosis with drug sensitivity.

### Statistical analysis

All statistical analyses were performed using R software (4.0.2). Independent-samples t-test was carried out to quantify the analysis of variables; ROC curves and Kaplan-Meier survival analysis were employed to predict the accuracy of the model; uni- and multi-Cox regression confirmed the independent prognostic value of the model. The subgroups with different clinical characteristics were investigated to fully evaluate the stability of risk characteristics, and Student’s t-test and Wilcoxon signed-rank test were conducted to explore the differences between these subgroups. The result p<0.05 indicated that all analyses had a statistical meaning.

## Results

### arlncRNA in bladder cancer patients

**Figure 1:**
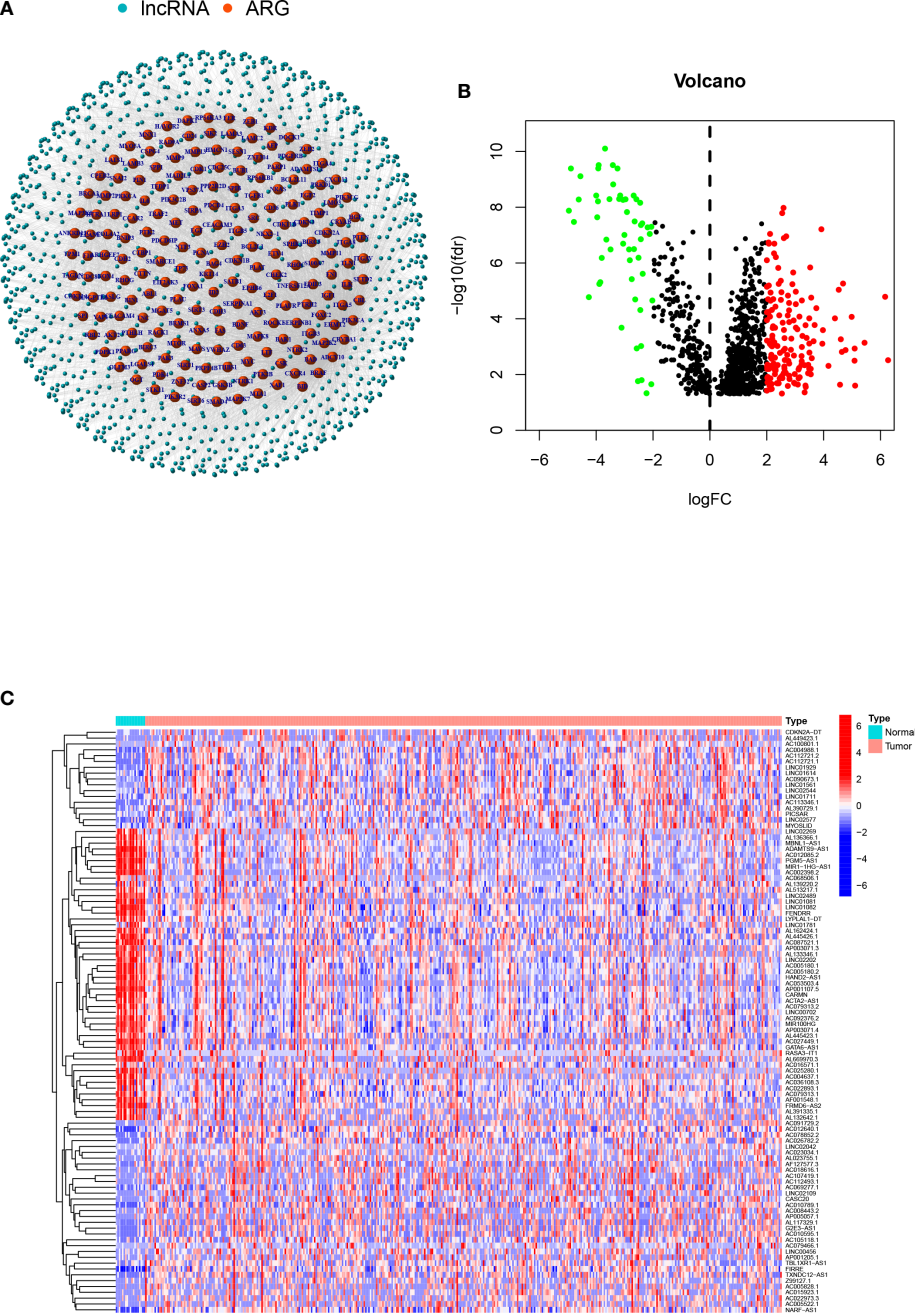
arlncRNAs identification and expression in bladder cancer: **(A)** arlncRNAs-ARGS network diagram; **(B)** Volcano plot of Log2 fold change (FC)>2 differentially-expressed ARGs; **(C)** Volcano plot of 101 arlncRNAs in normal and tumor samples.

A total of 1109 arlncRNAs were obtained *via* co-expression analysis, and their network relationship with ARG was mapped using expression data (**Figure 1A**). Differential analysis performed with the criterion of (|Log2FC|>2 and p<0.05) suggested that 169 genes were upregulated and 54 downregulated in expression (**Figure 1B**), and the heat map displayed the top 100 genes among |Log2FC| values (**Figure 1C**).

### Establishment of the model

**Figure 2:**
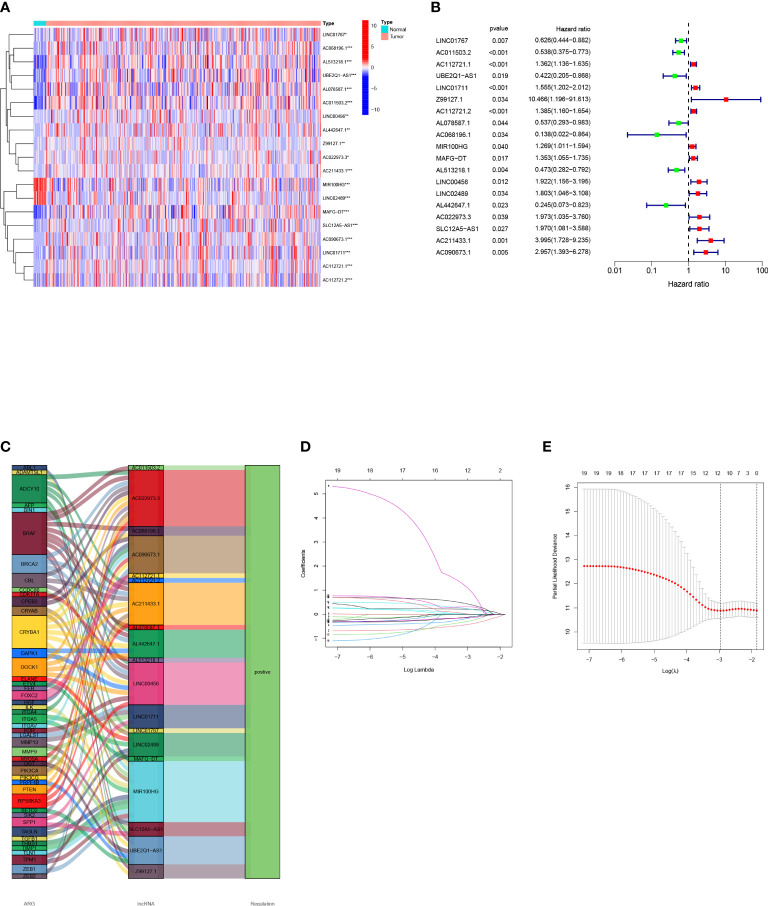
Construction of a bladder cancer arlncRNAs risk model: **(A)** Volcano plot of 19 prognosis-related arlncRNAs expressed in normal tissues and tumors; **(B)** Forest map of prognostic arlncRNAs extracted by uni-COX regression; **(C)** Sankey diagram of ARGs-arlncRNAs correlation; **(D)** Screening of prognostic arlncRNAs *via* cross validation; **(E)** Curve of error rate 10-fold cross-validation.

First, we obtained 19 OS-related arlncRNAs from uni-COX regression, and drew a heat map and forest map to show their expression (**Figure 2A, B**). The Sankey diagram indicated that the expression of these lncRNAs was upregulated (**Figure 2C**). We got seven lncRNAs after LASSO analysis and used them to build a risk model according to the following risk score formula: Risk score=LINC01767×(0.287716362819996)+AC011503.2×(-0.760808745099926)+`UBE2Q1-AS1`×(-0.692732045605004)+Z99127.1×(2.38512933255853)+AC112721.2×(0.296202121517627)+`MAFG-DT`×(0.458708109689086)+LINC00456×(0.820151733094388) (**Figure 2D, E**).

### Validation and application of the model

**Figure 3:**
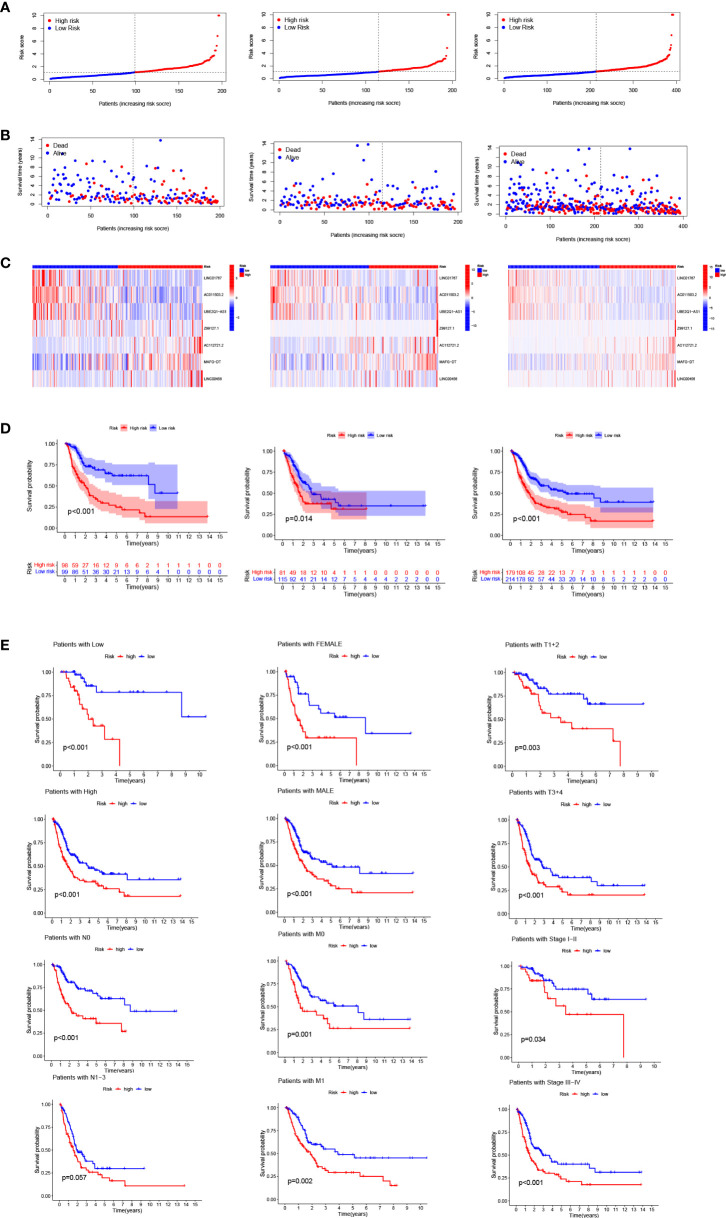
Risk model prognosis in the train, test and entire sets: **(A)** Risk score distribution diagram of train, test and entire sets; **(B)** Heat map of seven prognosis-related arlncRNAs; **(C)** The heat map of 7 lncRNAs expression in the train, test, and entire sets, respectively. **(D)** Survival analysis of high- and low-risk patients in train, test, and entire sets; **(E)** Survival analysis of clinicopathologic characteristics of high- and low-risk patients in entire sets.

**Figure 4:**
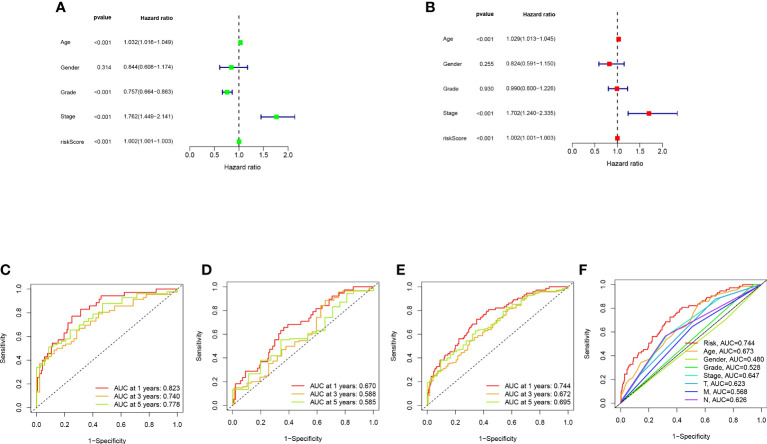
Validation of model assessment: **(A)** Uni- and multi-Cox analyses of clinical features; **(C-E)** 1-, 3-, and 5-year ROC curves of train, test, and entire sets; **(F)** ROC curves of risk scores and other clinical features.

Bladder cancer patients were randomly grouped as test and train, which were further classified into two groups (i.e., high-risk and low-risk) by median risk score. The risk score distribution, survival status and survival time expression results of the patients in the train group, the test group and as a whole were evaluated by this model formula. The result showed that the OS was much higher in the low-risk group than in the high-risk group (**Figure 3A-D**), so was other clinical characteristics like TNM stage, grade, age and gender (**Figure 3E**). To verify model accuracy, we performed uni- and multi-COX regression. The risk ratios (HR) and 95% confidence interval (CI) were 1.002 and 1.001-1.003 (p<0.001) respectively through uni-COX analysis, and 1.002 and 1.001-1.003 (p<0.001) respectively (**Figure 4A, B**) through multi-COX analysis, indicating that this model can be used as an independent prognosis factor. AUCs were 0.744, 0.672 and 0.695 (**Figure 4C-F**) respectively in one, three and five years. The model had a score of 0.744, representing high sensitivity and specificity.

### Establishment of nomogram

**Figure 5:**
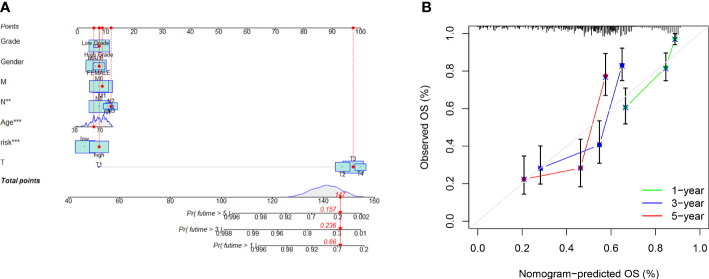
Risk model nomogram construction and verification: **(A)** Nomogram for predication of 1-, 3-, and 5- year survival rates; **(B)** Calibration curves for prediction of 1-, 3-, and 5-year survival rates.

We created a nomogram (**Figure 5A**) using the clinical factors and risk scores to further study the predictive power of the model, which was able to predict the morbidity of one-, three-, and five-year OS. The calibration diagram exhibited good agreement between the nomogram and the model (**Figure 5B**).

### Functional analysis of the model

**Figure 6:**
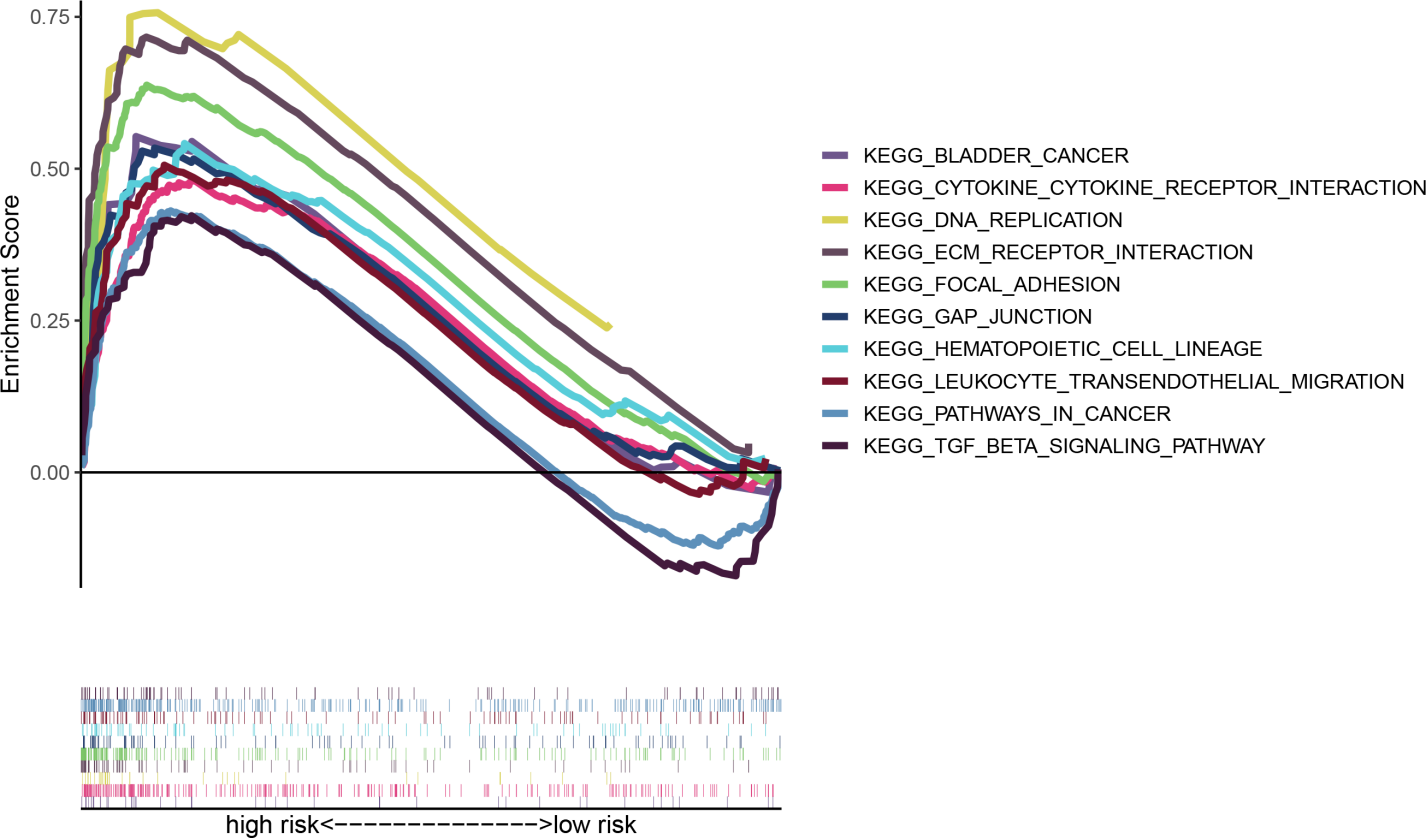
Multi-GSEA analysis of high- and low-risk patients in the risk model.

GSEA analysis reveals that the enrichment pathways of the high-risk group include DNA replication, focal adhesion, TGF, bladder tumor signaling pathway, extracellular matrix (ECM) receptor pathway, and the like (**Figure 6**). ECM has been reported to constitute a scaffold for TME and to regulate cancer behaviors (20, 21). The TGF signaling pathway is able to suppress tumors, including cell cycle arrest and apoptosis (22). There are also immune-related pathways such as leukocyte transendothelial migration. So, our purpose was to do immune-associated researches based on the risk model.

### Immunological characteristics of risk groups and exploration of clinical treatment

**Figure 7:**
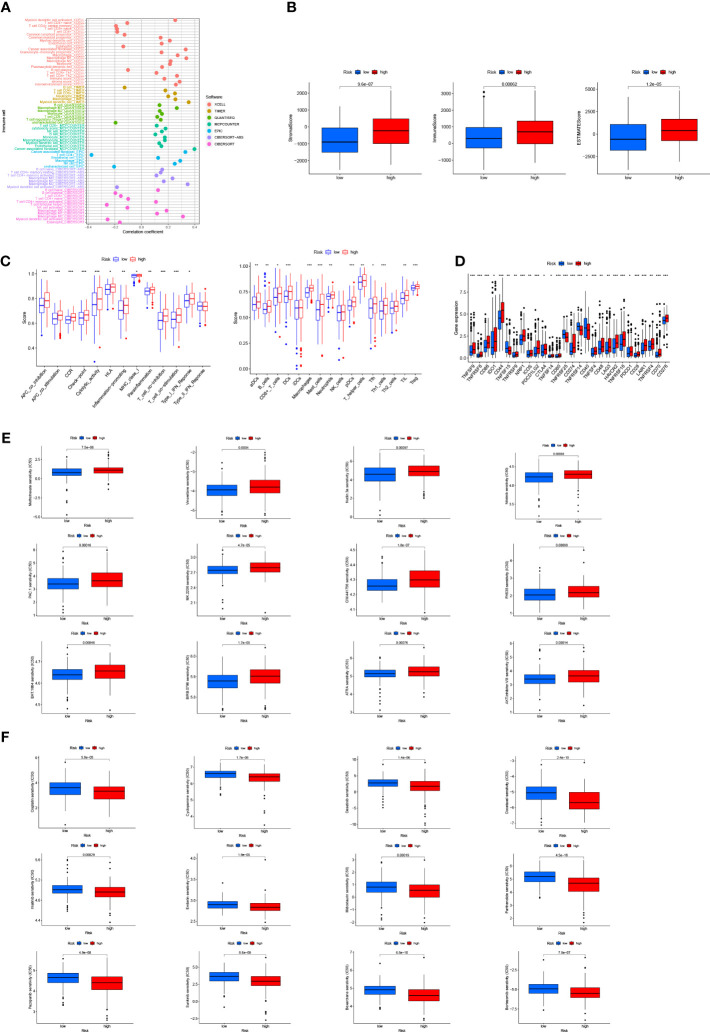
Discussion on immune infiltration and immunotherapy for patients with bladder cancer: **(A)** Bubble chart for immune cell correlation analysis; **(B)** Differences in immune microenvironment scores of patients at high and low risks; **(C)** Boxplots of immune cells in high- and low-risk groups and immune-related pathway analysis; **(D)** Analysis of immune checkpoints of the two risk groups; **(E, F)** Analysis of patients’ sensitivity to drugs. ***p < 0.001, **p < 0.01, *p < 0.05.

We investigated the correlation between risk score and tumor-infiltrating immune cells (**Figure 7A**), and the results showed a closer relationship between the immune cell and the high risk group on different platforms [e.g., common myeloid progenitor, myeloid dendritic cell, macrophage M2 in XCELL platform, T cells CD8+, CD4+ in TIMER platform, Endothelial cells in EPIC platform, and macrophage M1, M2 cells (**Supplementary Table S3**) in CIBERSORT-ABS platform]. According to TME scores, the high-risk group outstripped the low-risk group in terms of interstitial cystitis (IC), immunization and assessment (**Figure 7B**). According to ssGSEA (**Figure 7C**) analysis, the proportion of immune cell subpopulations and the component levels and functions of relevant pathways rose notably in almost all high-risk groups, where the vast majority of immune checkpoints showed a greater degree of activation (**Figure 7D**). The above results suggest that the high-risk group had higher immune infiltration than the lower one. This implies that we can select more appropriate immunosuppressives. It can be seen from the two groups’ drug susceptibility researched according to the “pRophetic” R package that the low-risk group was more sensitive to Methotrexate, Vinorelbine, Nutlin and Nilotinib (**Figure 7E**) while the high-risk group was more sensitive to Cisplatin, Cyclopamine, Docetaxel, Dasatinib and Imatinib (**Figure 7F**). It is a valuable guidance for the medication of bladder cancer patients.

### Immunological characteristics of different bladder cancer subtypes and prospects for treatment

**Figure 8:**
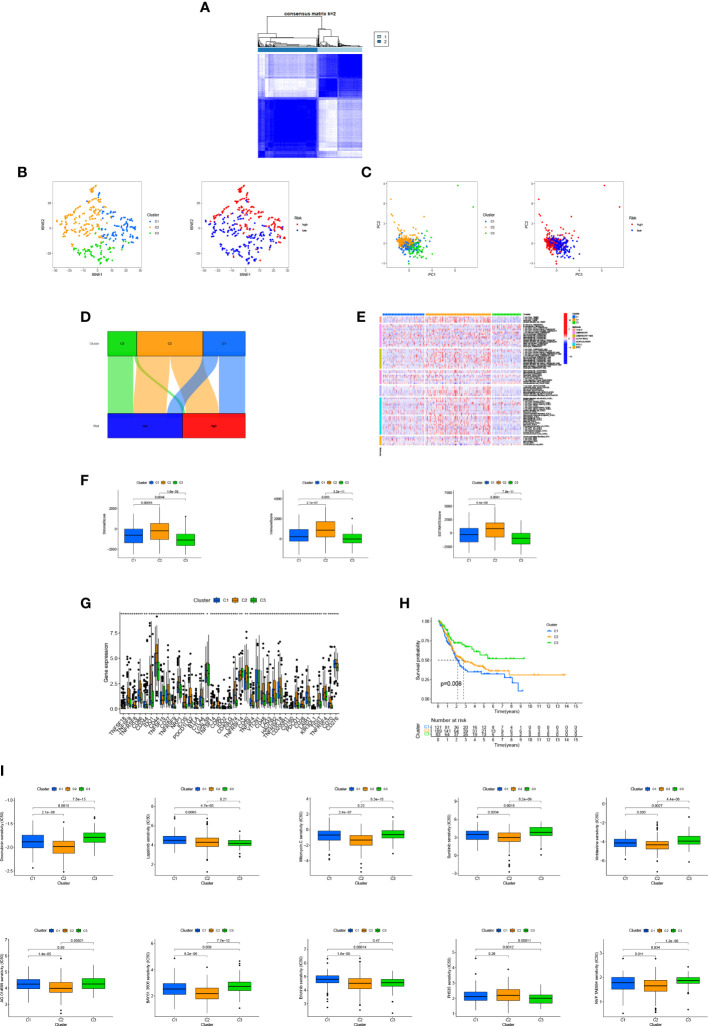
TME differences and underlying immunotherapy of different subtypes of bladder cancer: **(A)** Division of bladder cancer into three subtypes; **(B, C)** The t-SNE and PCA analysis of different subtypes; **(D)** Sankey diagram of interrelationship between three subtypes and high- and low-risks; **(E)** Heat map of immunocyte-related analysis of subtypes; **(F)** Differences in immune microenvironment scores of different subtypes; **(G)** Histogram of immune checkpoint expression differences between subtypes; **(H)** OS analysis curves of patients with different subtypes of bladder cancer; **(I)** cluster 2 drug sensitivity analysis. **p < 0.01, *p < 0.05.

Based on arlncRNA expression, we split bladder cancer patients into three subtypes (**Figure 8A** and **Supplementary Figure S1**). T-SNE results show (**Figure 8B**) that the distribution of the three clusters and the two risk groups is clear. The PCA results imply (**Figure 8C**) clear accumulation characteristics in terms of distribution between the two risk groups and between the three subtypes. In the Sankey diagram (**Figure 8D**), substantially all of cluster3 are in the low-risk group, and clusters 1 and 2 are equally distributed in high- and low-risk groups. In the immune cell heat map (**Figure 8E**), cluster 2 has the greatest extent of immune cell infiltration on different platforms, takes the first place in the facets of IC score, immune score or assessment score (**Figure 8F**). Also, the majority of immune checkpoints including PD1, PD -L1, CD40 and CD70 are more active in cluster 2 than in cluster 1 and cluster 3 (**Figure 8G**). This suggests cluster 2 may have a potentially good immunotherapeutic effect. Survival analysis indicates that cluster 3 has the best survival time and cluster1 the worst (**Figure 8H**). Likewise, by comparing drug sensitivity, we found higher sensitivity to most common immunotherapeutic drugs in cluster 2, including, for instance, Doxorubicin, Sunitinib, Vinblastine, Mitomycin and Lapatinib, (**Figure 8I**), which is helpful for us to explore more precise immunotherapy.

## Discussion

Bladder cancer is characterized by insidious onset and being highly aggressive. The five-year OS of the patients with metastatic bladder cancer accounts only for 6% (23), and the important role anoikis plays in tumor spread, metastasis and angiogenesis has been proven by researches (24–26). Thus, we created an arlncRNAs-based model aiming to predict the prognosis of bladder cancer patients and open up the possibility of efficacious medication.

During the research, we gathered 1019 differentially expressed arlncRNAs, and identified those with a prognostic value. We performed uni- and multi-Cox regression and LASSO regression, whereby seven arlncRNAs were validated for the construction of risk models, including UBE2Q1-AS1 that constituted a prognostic model for gastric cancer (27), `MAFG-DT` which was also a crucial member of the prognostic model for breast cancer (28), and AC112721.2 that regulated breast cancer expression through the TGF-β signaling pathway (29). Further studying these lncRNAs may provide a new target for oncotherapy. By median risk score the patients were split into two categories: high risk and low risk. We validated the predictive accuracy of the risk model with the aid of ROC curves, and plotted a nomogram predicting the prognosis of bladder cancer patients. The results predicted by the model were identical to the outcomes predicted by the nomogram. Similarly, clinicopathological analysis and survival analysis demonstrated that the model was highly sensitive to survival prediction. According to Cox regression, the model might be an independent factor of prognostic implications.

GSEA results disclose that the high-risk group was enriched in cancer signaling, DNA replication, and immune-related pathways. Exploration of differences in immune microenvironment between the patients at low and high risks, examination of immune checkpoints, TME scores, and assessment of immune state by the ssGSEA method revealed a higher level of immune infiltration in the high-risk group; some researches indicated that the level of immune infiltration was highly correlated with the efficacy of immunotherapy (30, 31). And we also probed into the sensitivity of immune drugs to both groups, offering new ideas and options for bladder cancer immunotherapy.

By expression, arlncRNAs were divided *via* consensus clustering into three subgroups. PCA analysis indicates that each cluster had its inherent aggregation characteristics; according to the results of Kaplan-Meier analysis, cluster 3 had a better prognosis; immune checkpoint results suggested that PD1, PD-L1 were most active in cluster 2, implying that cluster 2 was probably more effective against this type of immune checkpoint inhibitors and had a higher TME score. All this proves that cluster 2 had the highest extent of immune infiltration and a higher sensitivity to immunotherapy. What’s more, drug susceptibility shed a light on clinical medication for different subtypes of bladder cancer patients.

Still, we need to fill up some deficiencies. Firstly, the data used to validate the model are all downloaded from TCGA instead of ours. Secondly, further verification and exploration of biological functions are not yet done for the pathways in the present research and associated lncRNAs. These drawbacks will be included in our follow-up plans and addressed.

All in all, we built a bladder cancer risk model of seven arlncRNAs, discovered the signaling pathways it might be involved in, and evaluated its accuracy in prognostic prediction, immune infiltration, and drug sensitivity. Also, we classified the bladder cancer patients into different subtypes and discussed the differences in TME and sensitivity to drugs between these subtypes. Our research findings provide new strategies for prognostic assessment and treatment of bladder cancer patients.

## Data availability statement

The original contributions presented in the study are included in the article/**Supplementary Materials**. Further inquiries can be directed to the corresponding authors.

## Author contributions

SDL, YYZ, and XWL conceived and devised the study, YYZ performed bioinformatic and statistical analysis. XDL, YGZ, TTZ, CC, JWL, Li-W, XJ, LW and ML found testify data and analysis tools. YYZ, XWL and SDL supervised research and wrote the manuscript. ML revised the manuscript. All authors contributed to the paper and approved the submitted version.

## Funding

This study was supported by The General Hospital of Western Theater Command (grant no. 2021-XZYG-A11) and the urology department of The General Hospital of WTC.

## Acknowledgments

We acknowledge TCGA database and our colleagues for their contributions and commitment on this paper.

## Conflict of interest

The authors declare that the research was conducted in the absence of any commercial or financial relationships that could be construed as a potential conflict of interest.

## Publisher’s note

All claims expressed in this article are solely those of the authors and do not necessarily represent those of their affiliated organizations, or those of the publisher, the editors and the reviewers. Any product that may be evaluated in this article, or claim that may be made by its manufacturer, is not guaranteed or endorsed by the publisher.

## Supplementary material

Supplementary Figure 1 The heat maps, cumulative distribution function (CDF) maps and consensus CDF maps for different subtypes

Supplementary Table 1 The Table of ARGs

Supplementary Table 2 The network data of ARGs and arlncRNAs

Supplementary Table 3 The profile of Immune cell infiltration in high- and low-risk groups at different platforms

## References

[1] Garg M. Urothelial cancer stem cells and epithelial plasticity: Current concepts and therapeutic implications in bladder cancer. Cancer Metastasis Rev (2015) 34(4):691–701. doi: 10.1007/s10555-015-9589-6 26328525

[2] Bersanelli M Buti S Giannatempo P Raggi D Necchi A Leonetti A. Outcome of patients with advanced upper tract urothelial carcinoma treated with immune checkpoint inhibitors: A systematic review and meta-analysis. Crit Rev Oncol Hematol (2021) 159:103241. doi: 10.1016/j.critrevonc.2021.103241 33545355

[3] Fang D Kitamura H. Cancer stem cells and epithelial-mesenchymal transition in urothelial carcinoma: Possible pathways and potential therapeutic approaches. Int J Urol (2018) 25(1):7–17. doi: 10.1111/iju.13404 28697535

[4] Prout GR Marshall VF. The prognosis with untreated bladder tumors. Cancer (1956) 9(3):551–8. doi: 10.1002/1097-0142(195605/06)9:3<551::aid-cncr2820090319>3.0.co;2-2 13330006

[5] Tran L Xiao JF Agarwal N Duex JE Theodorescu D. Advances in bladder cancer biology and therapy. Nat Rev Cancer (2021) 21(2):104–21. doi: 10.1038/s41568-020-00313-1 PMC10112195 33268841

[6] Kim YN Koo KH Sung JY Yun UJ Kim H. Anoikis resistance: An essential prerequisite for tumor metastasis. Int J Cell Biol (2012) 2012:306879. doi: 10.1155/2012/306879 22505926 PMC3296207

[7] Guadamillas MC Cerezo A Del Pozo MA. Overcoming anoikis–pathways to anchorage-independent growth in cancer. J Cell Sci (2011) 124(Pt 19):3189–97. doi: 10.1242/jcs.072165 21940791

[8] Cao Z Livas T Kyprianou N. Anoikis and emt: Lethal “Liaisons” during cancer progression. Crit Rev Oncog (2016) 21(3-4):155–68. doi: 10.1615/CritRevOncog.2016016955 PMC5451151 27915969

[9] Chen S Gu J Zhang Q Hu Y Ge Y. Development of biomarker signatures associated with anoikis to predict prognosis in endometrial carcinoma patients. J Oncol (2021) 2021:3375297. doi: 10.1155/2021/3375297 34992654 PMC8727165

[10] St Laurent G Wahlestedt C Kapranov P. The landscape of long noncoding rna classification. Trends Genet (2015) 31(5):239–51. doi: 10.1016/j.tig.2015.03.007 PMC4417002 25869999

[11] Robinson EK Covarrubias S Carpenter S. The how and why of lncrna function: An innate immune perspective. Biochim Biophys Acta Gene Regul Mech (2020) 1863(4):194419. doi: 10.1016/j.bbagrm.2019.194419 31487549 PMC7185634

[12] Bhan A Soleimani M Mandal SS. Long noncoding rna and cancer: A new paradigm. Cancer Res (2017) 77(15):3965–81. doi: 10.1158/0008-5472.Can-16-2634 PMC8330958 28701486

[13] Lv W Wang Y Zhao C Tan Y Xiong M Yi Y. Identification and validation of M6a-related lncrna signature as potential predictive biomarkers in breast cancer. Front Oncol (2021) 11:745719. doi: 10.3389/fonc.2021.745719 34722303 PMC8555664

[14] Liu L Huang L Chen W Zhang G Li Y Wu Y. Comprehensive analysis of necroptosis-related long noncoding rna immune infiltration and prediction of prognosis in patients with colon cancer. Front Mol Biosci (2022) 9:811269. doi: 10.3389/fmolb.2022.811269 35237659 PMC8883231

[15] Lu Y Luo X Wang Q Chen J Zhang X Li Y. A novel necroptosis-related lncrna signature predicts the prognosis of lung adenocarcinoma. Front Genet (2022) 13:862741. doi: 10.3389/fgene.2022.862741 35368663 PMC8969905

[16] Deng Z Li X Shi Y Lu Y Yao W Wang J. A novel autophagy-related incrnas signature for prognostic prediction and clinical value in patients with pancreatic cancer. Front Cell Dev Biol (2020) 8:606817. doi: 10.3389/fcell.2020.606817 33384999 PMC7769875

[17] Meng T Huang R Zeng Z Huang Z Yin H Jiao C. Identification of prognostic and metastatic alternative splicing signatures in kidney renal clear cell carcinoma. Front Bioeng Biotechnol (2019) 7:270. doi: 10.3389/fbioe.2019.00270 31681747 PMC6803439

[18] Bunea F She Y Ombao H Gongvatana A Devlin K Cohen R. Penalized least squares regression methods and applications to neuroimaging. Neuroimage (2011) 55(4):1519–27. doi: 10.1016/j.neuroimage.2010.12.028 PMC5485905 21167288

[19] Hong W Liang L Gu Y Qi Z Qiu H Yang X. Immune-related lncrna to construct novel signature and predict the immune landscape of human hepatocellular carcinoma. Mol Ther Nucleic Acids (2020) 22:937–47. doi: 10.1016/j.omtn.2020.10.002 PMC7670249 33251044

[20] Jang I Beningo KA. Integrins, cafs and mechanical forces in the progression of cancer. Cancers (Basel) (2019) 11(5), 721. doi: 10.3390/cancers11050721 31137693 PMC6562616

[21] Pickup MW Mouw JK Weaver VM. The extracellular matrix modulates the hallmarks of cancer. EMBO Rep (2014) 15(12):1243–53. doi: 10.15252/embr.201439246 PMC4264927 25381661

[22] Colak S Ten Dijke P. Targeting tgf-β signaling in cancer. Trends Cancer (2017) 3(1):56–71. doi: 10.1016/j.trecan.2016.11.008 28718426

[23] Liu D Qiu X Xiong X Chen X Pan F. Current updates on the role of reactive oxygen species in bladder cancer pathogenesis and therapeutics. Clin Transl Oncol (2020) 22(10):1687–97. doi: 10.1007/s12094-020-02330-w PMC7423792 32189139

[24] Frisch SM Ruoslahti E. Integrins and anoikis. Curr Opin Cell Biol (1997) 9(5):701–6. doi: 10.1016/s0955-0674(97)80124-x 9330874

[25] Tan K Goldstein D Crowe P Yang JL. Uncovering a key to the process of metastasis in human cancers: A review of critical regulators of anoikis. J Cancer Res Clin Oncol (2013) 139(11):1795–805. doi: 10.1007/s00432-013-1482-5 PMC11824334 23912151

[26] Sakamoto S McCann RO Dhir R Kyprianou N. Talin1 promotes tumor invasion and metastasis *Via* focal adhesion signaling and anoikis resistance. Cancer Res (2010) 70(5):1885–95. doi: 10.1158/0008-5472.Can-09-2833 PMC2836205 20160039

[27] Zhang X Jiang Y Xie Y Leng X Song F. Comprehensive analysis of lncrnas associated with the pathogenesis and prognosis of gastric cancer. DNA Cell Biol (2020) 39(2):299–309. doi: 10.1089/dna.2019.5161 31934786

[28] Su X Yu Z Zhang Y Chen J Wei L Sun L. Construction and analysis of the dysregulated cerna network and identification of risk long noncoding rnas in breast cancer. Front Genet (2021) 12:664393. doi: 10.3389/fgene.2021.664393 34149805 PMC8212960

[29] Vishnubalaji R Alajez NM. Epigenetic regulation of triple negative breast cancer (Tnbc) by tgf-β signaling. Sci Rep (2021) 11(1):15410. doi: 10.1038/s41598-021-94514-9 34326372 PMC8322425

[30] Ahluwalia P Ahluwalia M Mondal AK Sahajpal N Kota V Rojiani MV. Immunogenomic gene signature of cell-death associated genes with prognostic implications in lung cancer. Cancers (Basel) (2021) 13(1), 155. doi: 10.3390/cancers13010155 33466402 PMC7795632

[31] Hu FF Liu CJ Liu LL Zhang Q Guo AY. Expression profile of immune checkpoint genes and their roles in predicting immunotherapy response. Brief Bioinform (2021) 22(3), bbaa176. doi: 10.1093/bib/bbaa176 32814346

