# Supplementary figures and images for: Comprehensive analysis of anoikis-related long non-coding RNA immune infiltration in patients with bladder cancer and immunotherapy
Source: Front Immunol. 2022 Nov 25;13:1055304. doi: 10.3389/fimmu.2022.1055304 (PMC9732092; doi:10.3389/fimmu.2022.1055304)

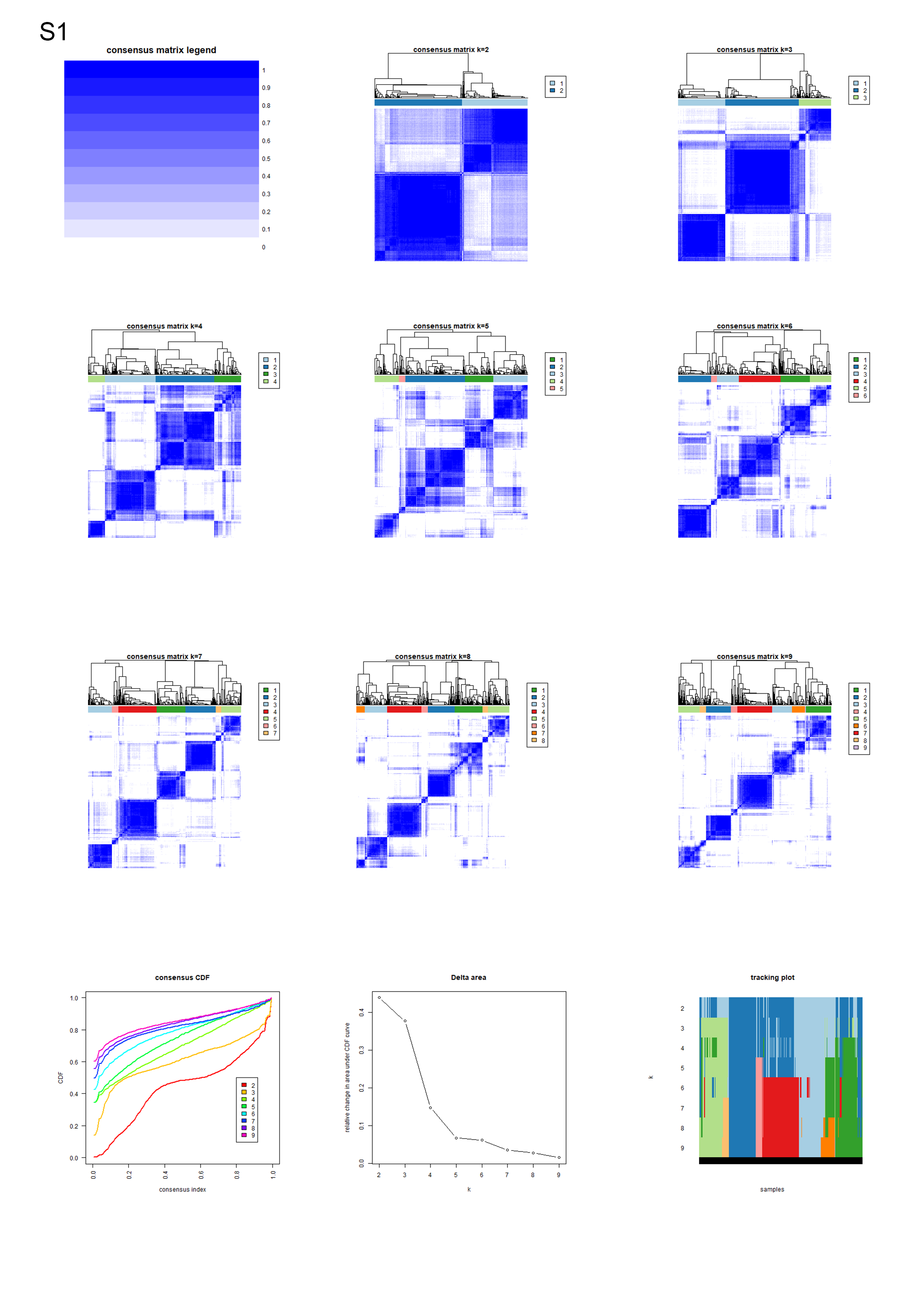

Supplement: Supplementary Figure 1 — The heat maps, cumulative distribution function (CDF) maps and consensus CDF maps for different subtypes [file Image_1.tif]
