# Supplementary material for: Comprehensive analysis of anoikis-related long non-coding RNA immune infiltration in patients with bladder cancer and immunotherapy
Source: Front Immunol. 2022 Nov 25;13:1055304. doi: 10.3389/fimmu.2022.1055304 (PMC9732092; doi:10.3389/fimmu.2022.1055304)
Supplement: Supplementary Table 2 — The network data of ARGs and arlncRNAs [file Table_2.docx]

| id | coef |
| --- | --- |
| LINC01767 | -0.28772 |
| AC011503.2 | -0.76081 |
| `UBE2Q1-AS1` | -0.69273 |
| Z99127.1 | 2.385129 |
| AC112721.2 | 0.296202 |
| `MAFG-DT` | 0.458708 |
| LINC00456 | 0.820152 |
