# Supplementary material for: Comprehensive analysis of anoikis-related long non-coding RNA immune infiltration in patients with bladder cancer and immunotherapy
Source: Front Immunol. 2022 Nov 25;13:1055304. doi: 10.3389/fimmu.2022.1055304 (PMC9732092; doi:10.3389/fimmu.2022.1055304)
Supplement: Supplementary Table 3 — The profile of Immune cell infiltration in high- and low-risk groups at different platforms [file Table_3.docx]

| immune | cor | pvalue |
| --- | --- | --- |
| B cell_TIMER | -0.12682 | 0.0118582 |
| T cell CD4+_TIMER | 0.179172 | 0.0003576 |
| T cell CD8+_TIMER | 0.251117 | 0.0000005 |
| Neutrophil_TIMER | 0.200702 | 0.0000615 |
| Macrophage_TIMER | 0.254753 | 0.0000003 |
| Myeloid dendritic cell_TIMER | 0.354131 | 0.0000000 |
| B cell naive_CIBERSORT | 0.124116 | 0.0138092 |
| B cell plasma_CIBERSORT | -0.19644 | 0.0000885 |
| T cell CD8+_CIBERSORT | -0.15516 | 0.0020366 |
| T cell CD4+ naive_CIBERSORT | -0.10023 | 0.0470740 |
| T cell CD4+ memory activated_CIBERSORT | 0.108766 | 0.0311052 |
| T cell follicular helper_CIBERSORT | -0.26054 | 0.0000002 |
| NK cell activated_CIBERSORT | -0.10575 | 0.0361108 |
| Macrophage M0_CIBERSORT | 0.188109 | 0.0001763 |
| Macrophage M1_CIBERSORT | 0.1169 | 0.0204479 |
| Macrophage M2_CIBERSORT | 0.311 | 0.0000000 |
| Myeloid dendritic cell activated_CIBERSORT | -0.25448 | 0.0000003 |
| Eosinophil_CIBERSORT | -0.16296 | 0.0011868 |
| B cell naive_CIBERSORT-ABS | 0.15536 | 0.0020092 |
| T cell CD4+ memory resting_CIBERSORT-ABS | 0.144002 | 0.0042291 |
| T cell CD4+ memory activated_CIBERSORT-ABS | 0.112175 | 0.0261670 |
| Macrophage M0_CIBERSORT-ABS | 0.232835 | 0.0000031 |
| Macrophage M1_CIBERSORT-ABS | 0.167032 | 0.0008870 |
| Macrophage M2_CIBERSORT-ABS | 0.346373 | 0.0000000 |
| Myeloid dendritic cell activated_CIBERSORT-ABS | -0.1843 | 0.0002392 |
| B cell_QUANTISEQ | 0.135219 | 0.0072656 |
| Macrophage M1_QUANTISEQ | 0.264921 | 0.0000001 |
| Macrophage M2_QUANTISEQ | 0.136958 | 0.0065426 |
| Monocyte_QUANTISEQ | 0.153454 | 0.0022846 |
| T cell CD8+_QUANTISEQ | 0.118035 | 0.0192480 |
| T cell regulatory (Tregs)_QUANTISEQ | 0.144557 | 0.0040827 |
| uncharacterized cell_QUANTISEQ | -0.19461 | 0.0001032 |
| T cell CD8+_MCPCOUNTER | 0.114092 | 0.0236987 |
| cytotoxicity score_MCPCOUNTER | 0.180273 | 0.0003283 |
| NK cell_MCPCOUNTER | 0.152702 | 0.0024024 |
| B cell_MCPCOUNTER | 0.10409 | 0.0391570 |
| Monocyte_MCPCOUNTER | 0.175076 | 0.0004891 |
| Macrophage/Monocyte_MCPCOUNTER | 0.175076 | 0.0004891 |
| Myeloid dendritic cell_MCPCOUNTER | 0.133039 | 0.0082727 |
| Endothelial cell_MCPCOUNTER | 0.149352 | 0.0029973 |
| Cancer associated fibroblast_MCPCOUNTER | 0.397477 | 0.0000000 |
| Myeloid dendritic cell activated_XCELL | 0.253464 | 0.0000004 |
| T cell CD4+ naive_XCELL | -0.10667 | 0.0345235 |
| T cell CD4+ central memory_XCELL | -0.19156 | 0.0001330 |
| T cell CD8+ naive_XCELL | -0.18414 | 0.0002423 |
| T cell CD8+_XCELL | -0.18226 | 0.0002810 |
| Common lymphoid progenitor_XCELL | -0.12714 | 0.0116459 |
| Common myeloid progenitor_XCELL | 0.14354 | 0.0043543 |
| Myeloid dendritic cell_XCELL | 0.21277 | 0.0000211 |
| Endothelial cell_XCELL | 0.151269 | 0.0026423 |
| Eosinophil_XCELL | -0.13694 | 0.0065517 |
| Cancer associated fibroblast_XCELL | 0.331838 | 0.0000000 |
| Granulocyte-monocyte progenitor_XCELL | 0.150373 | 0.0028032 |
| Macrophage_XCELL | 0.271244 | 0.0000000 |
| Macrophage M1_XCELL | 0.33657 | 0.0000000 |
| Macrophage M2_XCELL | 0.218648 | 0.0000122 |
| Monocyte_XCELL | 0.214174 | 0.0000185 |
| Plasmacytoid dendritic cell_XCELL | 0.154374 | 0.0021476 |
| B cell plasma_XCELL | -0.1013 | 0.0447524 |
| T cell CD4+ Th1_XCELL | 0.112242 | 0.0260775 |
| T cell CD4+ Th2_XCELL | 0.26627 | 0.0000001 |
| immune score_XCELL | 0.171756 | 0.0006273 |
| stroma score_XCELL | 0.285867 | 0.0000000 |
| microenvironment score_XCELL | 0.257985 | 0.0000002 |
| Cancer associated fibroblast_EPIC | 0.329184 | 0.0000000 |
| T cell CD4+_EPIC | -0.37577 | 0.0000000 |
| Endothelial cell_EPIC | 0.124867 | 0.0132417 |
| Macrophage_EPIC | 0.290339 | 0.0000000 |
| NK cell_EPIC | 0.249522 | 0.0000005 |
| uncharacterized cell_EPIC | -0.20581 | 0.0000394 |
